# Current Treatment of Drug-Resistant Tuberculosis in Children

**DOI:** 10.1007/s12098-023-04888-z

**Published:** 2023-11-23

**Authors:** H. Simon Schaaf, Jennifer Hughes

**Affiliations:** Desmond Tutu TB Centre, Department of Pediatrics and Child Health, Faculty of Medicine and Health Sciences, Stellenbosch University, Cape Town, South Africa

**Keywords:** Rifampicin-resistant, Multidrug-resistant, Tuberculosis, Treatment, Children

## Abstract

Optimal diagnosis and management of children aged <15 y with rifampicin- or multidrug-resistant tuberculosis (RR/MDR-TB) relies on identification of adults with the disease and pro-active screening of their close contacts. Children may be diagnosed with RR/MDR-TB based on microbiological confirmation from clinical specimens (sputum, gastric washings, stool), but usually the diagnosis is presumptive, with a history of exposure to RR/MDR-TB and clinical/radiological signs and symptoms suggestive of TB disease. RR/MDR-TB should also be considered in children where first-line TB treatment fails despite good adherence to therapy. Composition and duration of all-oral RR/MDR-TB treatment regimens in children are based on site and severity of TB disease, drug resistance profile of the *Mycobacterium tuberculosis* strain (isolated from the child or from the most likely source patient), inclusion of at least four drugs considered to be effective (with priority given to World Health Organization Group A and B drugs), toxicity and tolerability of medications (and feasibility of adverse effect monitoring in the child’s setting), and availability of child-friendly formulations of TB medications. Individualized RR/MDR-TB regimens are preferable to the standardised 9–12-mo regimen for children, and injectable agents must not be used. Optimal adherence to treatment relies on education, training and support for caregivers and others who are responsible for administering medications to children, as well as close clinical monitoring and early management of adverse effects. Children who are initiated on adequate RR/MDR-TB regimens have high treatment success rates, but efforts to find and treat more children with undiagnosed RR/MDR-TB are crucial to reduce childhood TB mortality.

## Introduction

An estimated 1.17 million children developed tuberculosis (TB) in 2021, of whom an estimated 25,000–32,000 had rifampicin-resistant (RR)-TB [1, 2]. RR-TB includes: rifampicin mono-resistant (RMR)-TB; multidrug-resistant (MDR)-TB, defined as disease caused by *Mycobacterium tuberculosis* resistant to both isoniazid and rifampicin with or without resistance to other antituberculosis drugs; pre-extensively drug-resistant (pre-XDR-TB), defined as MDR-TB with resistance to fluoroquinolones; and XDR-TB, defined as pre-XDR-TB with resistance to either bedaquiline and/or linezolid [3].

Most children (<15 y of age) with RR/MDR-TB are infected and develop disease following close contact with an infectious RR/MDR-TB source patient, usually an adult; however, older children and adolescents with adult-type, high bacillary load pulmonary TB may acquire RR/MDR-TB due to poor clinical management or poor treatment adherence for drug-susceptible TB [4].

## Diagnosing RR/MDR-TB in Children

The diagnosis of TB disease in children is usually based on patient history and clinical examination, with or without positive tests of *M. tuberculosis* infection, radiological imaging and microbiological tests for *M. tuberculosis*. Children with TB either present with clinical signs and symptoms of TB disease (passive case finding – often with more severe disease) or they are traced as contacts of infectious TB patients (active case finding – mainly with non-severe disease). RR/MDR-TB should be considered in children with recent RR/MDR-TB exposure or failure of first-line TB treatment despite good treatment adherence. Occasionally, children may have microbiological confirmation of RR/MDR-TB and may even be the index RR/MDR-TB patient (i.e., first diagnosed case) in a household or congregate setting. Microbiological testing for RR/MDR-TB includes mycobacterial culture and phenotypic drug susceptibility testing (DST), genotypic nucleic acid amplification tests (NAAT; e.g., Xpert MTB/RIF or TrueNat) or *M. tuberculosis* whole genome sequencing or targeted next generation sequencing with identification of mutations causing resistance [4]. Obtaining suitable clinical specimens for microbiological confirmation of TB can be challenging in children and microbiological tests are often negative due to the paucibacillary nature of the disease in most children. Therefore, children diagnosed with TB who have a history of contact with an infectious RR/MDR-TB patient (presumed RR/MDR-TB) or in whom first-line TB treatment has failed despite appropriate adherence (possible RR/MDR-TB) should receive empirical treatment for RR/MDR-TB, taking into account the DST result of the *M. tuberculosis* strain from the known source patient where available [4, 5].

Every effort should be made to obtain clinical specimens for microbiological confirmation and DST in children with presumed or possible RR/MDR-TB, preferably before starting any TB treatment. Although there is high concordance in results of DST, especially for rifampicin and isoniazid, between *M. tuberculosis* isolates from source patients and their household contacts [6], some children may be infected from other source patients, who may have drug-susceptible or more extensively resistant RR/MDR-TB, especially in high TB-burden settings. However, in children with TB disease and a known RR/MDR-TB source patient, appropriate empirical treatment should not be delayed in the absence of microbiological confirmation or while DST results are pending, especially as young children may rapidly progress to severe and disseminated disease without treatment.

## General Principles and Considerations in Treating Children with RR/MDR-TB

The general approach to treatment of RR/MDR-TB is the same for children of all ages (0–14 y) and is mainly based on determining the resistance profile of the infecting organism as well as the site and severity of the disease, although the composition and duration of treatment regimens may vary between individuals. The following factors should be considered:

1. The drug resistance profile of the *M. tuberculosis* strain isolated from the child, or from the most likely source patient if the child does not have an *M. tuberculosis* isolate of their own, will determine the composition, and possibly duration, of the treatment regimen [4]. Following up on all sequential DST results of RR/MDR-TB specimens from the child and/or the source patient is therefore essential, particularly to identify early any resistance to fluoroquinolones, bedaquiline, linezolid and/or clofazimine. Previous failed antituberculosis treatment, either in the child or in the known source case, as well as previous use of long or multiple courses of antibiotics (particularly fluoroquinolones), may influence the likely effectiveness of those drugs, due to the risk of acquired drug resistance [7, 8]. If no DST results are available for *M. tuberculosis* isolates from children in whom first-line therapy is failing, despite good adherence, the most likely *M. tuberculosis* resistance profile in the relevant geographic area should be considered when designing an appropriate empirical regimen [9].
2. Treatment regimens should include at least 4 drugs considered to be effective; WHO Group A and B drugs and delamanid are prioritised (Table 1) [10–14]. A drug is considered effective if DST confirms susceptibility or, in the absence of DST, there is no evidence of previous treatment failure (in the child or source patient) with a regimen including that drug. Possible addition of a 5th drug may be necessary (e.g., severe disease, complex site of disease, ≤1 WHO group A drug included), but regimens containing ≥5 drugs generally serve to increase the toxicity profile without necessarily improving treatment efficacy [5].
3. All-oral regimens must be prioritised. The highly toxic and poorly tolerated injectable agents (amikacin, streptomycin, meropenem) should only be considered in children when available drug options to build an effective salvage regimen are severely limited, e.g. XDR-TB or MDR-TB treatment failure, and only if the infecting organism is confirmed to be susceptible to the injectable agent and frequent toxicity monitoring is feasible [5].
4. Treatment duration is based on disease severity, i.e., severe vs. non-severe disease, according to definitions used in the SHINE trial, a prospective, randomized-controlled study among children treated for drug-susceptible TB [15], and the latest WHO definition of non-severe disease in children aged <15 y [10]. Non-severe TB includes uncomplicated peripheral lymph node TB, intrathoracic lymph node TB without airway compression or obstruction, uncomplicated TB pleural effusion, and/or paucibacillary, non-cavitary disease confined to no more than one lobe of the lungs and without a miliary pattern. Children with non-severe RR/MDR-TB disease may benefit from shorter treatment duration with fewer drugs, e.g. four drugs for only 6–9 mo, whereas those with severe disease may require an initial regimen of five drugs and longer duration of treatment [5, 10].
5. Site of TB disease may affect treatment duration, and children with more extensive disease, particularly central nervous system (CNS), miliary, bone or pericardial TB, are likely to require five drugs in their initial regimen. Treatment of RR/MDR-TB meningitis, CNS tuberculomas or miliary TB (which almost always includes the CNS) [16] should be guided by drug penetration of the blood–brain barrier (Table 2) [13, 17, 18].
6. Toxicity and tolerability of medications, and feasibility of adverse effect monitoring, may determine regimen composition. Use of multiple QT-prolonging agents necessitates regular electrocardiogram monitoring, and linezolid carries a considerable toxicity risk, with stringent monitoring requirements (e.g., frequent blood draws to check full blood count and differential white cell count) which can be challenging for children and their families in some settings.
7. Child-friendly formulations of TB medications should be used whenever possible, many of which are available from the Global Drug Facility [12]. Alternatively, extemporaneous preparations of some drugs can be prepared monthly by a pharmacy specialist [19–21], or caregivers can be trained to cut or crush the adult formulation tablets, mix with water, milk or other locally available food, and administer the full volume of the mixture (Table 1) [11].

**Table 1:**
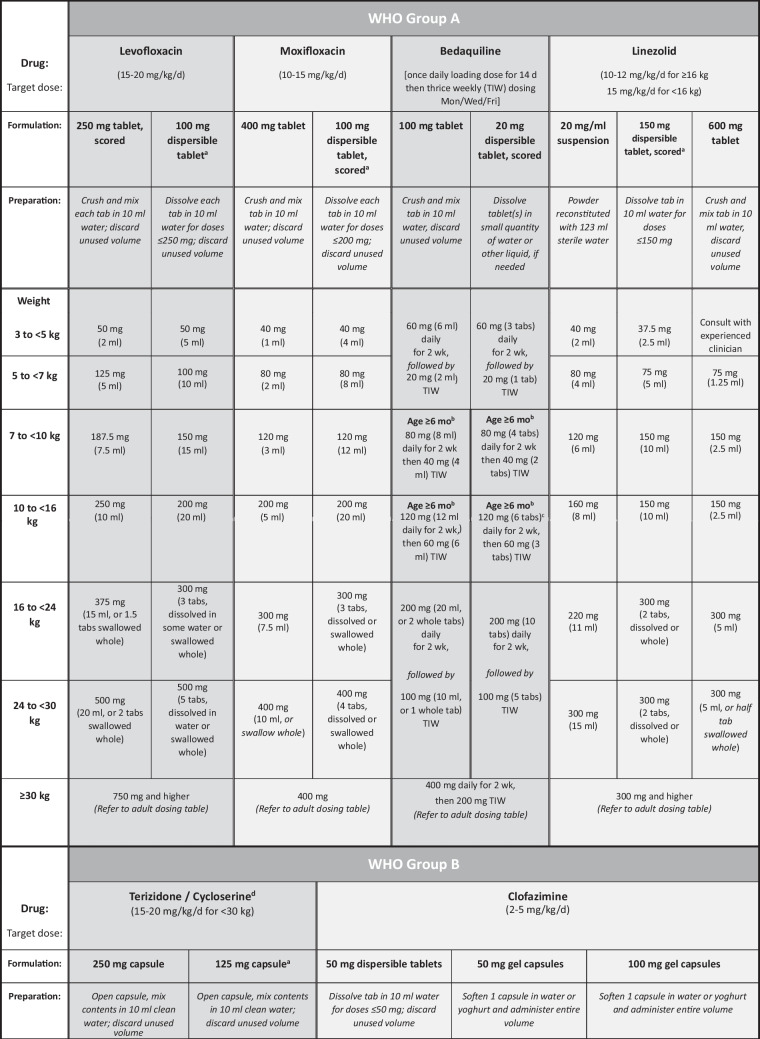

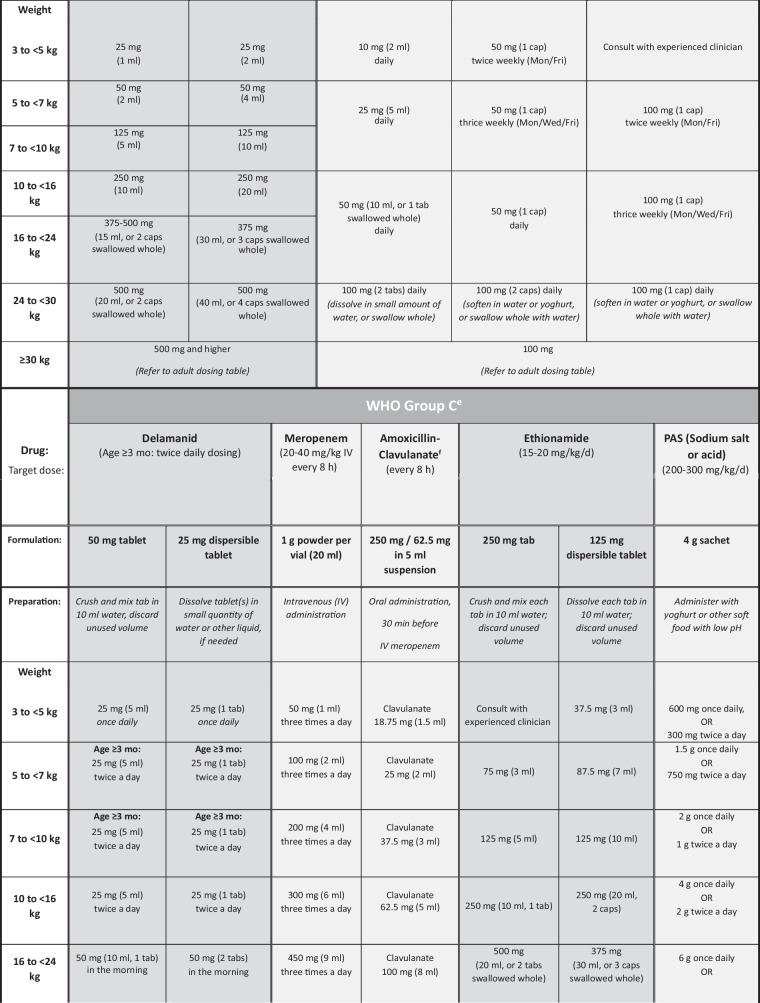

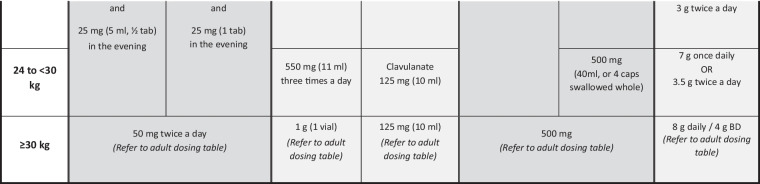
Weight- and age-based dosing recommendations for children and adolescents aged 3 mo to <15 y and weighing up to 30 kg according to World Health Organization classification (Groups A-C) of second-line antituberculosis drugs [10–12]

In general, for older children able to swallow tablets/capsules whole, avoid crushing and mixing tablets/capsules with water, as this may reduce palatability (tastes worse than swallowing tablets whole)

*BD* Twice a day, *cap* Capsule, *g* Grams, *kg* Kilograms, *mg* Milligrams, *ml* Milliliters, *Mon/Wed/Fri* Monday, Wednesday, Friday, *tab* Tablet, *TIW* Thrice weekly, *WHO* World Health Organization

^a^Refer to the Sentinel Dispersible Drug Information Sheets for more detailed dosing guidance based on narrower weight bands [12]

^b^Children aged 3–6 mo, bedaquiline dose is 60 mg daily for 2 wk then 20 mg Mon/Wed/Fri; switch to weight-based dosing after 6 mo of age

^c^Alternatively, give one 100 mg tablet and one 20 mg tablet of bedaquiline

^d^Do not co-administer high-dose isoniazid with terizidone/cycloserine as this may markedly reduce isoniazid concentrations [14]

^e^Pyrazinamide and ethambutol are part of Group C, but not included here (dosing is the same as for first-line TB regimens); note also that injectable aminoglycosides are not included here as these are not recommended for use in children and adolescents below 18 y of age, but if used as salvage therapy, calculate amikacin dose based on weight using 15–20 mg/kg/d (if given intramuscularly, add lidocaine to reduce pain [13])

^f^Amoxicillin-clavulanate is not categorised by WHO as a Group C drug but should always be co-administered with carbapenems

**Table 2:** World Health Organization classification (Groups A-C) of second-line antituberculosis drugs, important adverse effects, monitoring and cerebrospinal fluid penetration [17]

Drug group and drug name	Important adverse effects	Adverse effect monitoring	Cerebrospinal fluid (CSF) penetration
**Group A drugs**^**a**^			
Levofloxacin (Lfx)	Sleep disturbance, GI disturbance, arthralgia/ arthritis, raised intracranial pressure	Clinically	Good
Moxifloxacin (Mfx)	As for levofloxacin, plus QT-interval prolongation	Clinically; monthly ECG if given with other QT-prolonging drugs	Good
Bedaquiline (Bdq)	Headache, nausea, liver dysfunction, QT-interval prolongation	Clinically; monthly ECG if given with other QT-prolonging drugs	Does enter CSF – unbound fraction important
Linezolid (Lzd)	Diarrhea, nausea, headache, myelosuppression, peripheral neuropathy, optic neuritis, lactic acidosis and pancreatitis	Clinically; baseline 2-weekly FBC & differential WCC for first month, then monthly; visual acuity testing when possible	Good
**Group B drugs**			
Clofazimine (Cfz)	Skin discoloration, ichthyosis, abdominal pain, QTc prolongation	Clinically; monthly ECG if given with other QT-prolonging drugs	Poor
Cycloserine (Cs) /Terizidone (Trd)	Neurological and psychological effects	Clinically	Good
**Group C drugs**			
Ethambutol (E)	Optic neuritis	Clinically	Very poor
Delamanid (Dlm)	Nausea, vomiting, dizziness, paresthesia, anxiety, hallucinations, QTc prolongation	Clinically; monthly ECG if given with other QT-prolonging drugs	Likely enters CSF and good penetration in brain tissue
Pyrazinamide (Z)	Arthritis/arthralgia, hepatitis, skin rashes	Clinically; liver function tests if any vomiting or abdominal complaints	Good
Amikacin (Am)^b^ (Kanamycin and capreomycin same dose)	Ototoxicity (irreversible), nephrotoxicity *Use NOT recommended in children*	Monthly audiology and renal function testing, if no other options for treatment	Poor; moderate in acute inflammatory stage
Ethionamide (Eto) / Prothionamide (Pto)	GI disturbance, metallic taste, hypothyroidism	Clinically; thyroid function testing at least two-monthly	Good
Meropenem (Mpm)	GI intolerance, hypersensitivity reactions, seizures, liver and renal dysfunction	Clinically; liver and renal function testing	Good
Amoxicillin-clavulanate (Amx-Clv)	Only to be used with a carbapenem GI intolerance, hypersensitivity reactions	Clinically	Poor
*Para-*aminosalicylic acid (PAS)	GI intolerance, hypothyroidism, hepatitis	Clinically; thyroid function testing at least two-monthly	Moderate to poor. Use as single daily dose for better CSF penetration
Isoniazid high-dose (Hhd)	Hepatitis, peripheral neuropathy	Clinically; liver function tests if any vomiting or abdominal complaints	Good

*CSF* Cerebrospinal fluid, *ECG* Electrocardiogram, *FBC* Full blood count, *GI* Gastrointestinal, *WCC* White cell count

^a^WHO Group A drugs are considered highly effective and strongly recommended for inclusion in all regimens unless contra-indicated [18]

^b^Can be given with lidocaine to reduce pain of intramuscular injections [13]

## Recent Developments in RR/MDR-TB Treatment in Children

In March 2022, the WHO issued updated guidelines for the management of TB in children and adolescents, which recommend that bedaquiline and delamanid can be used in all age groups, along with updated dosing guidance for all TB drugs [10, 11]. Previous age restrictions on the use of these two medications in children are no longer relevant. Furthermore, the WHO now recommends 6-mo regimens of bedaquiline, pretomanid and linezolid, with or without moxifloxacin (BPaLM or BPaL), for non-pregnant persons aged 15 y and older for treating RR/MDR-TB [18]. As pretomanid dosing and safety has not yet been determined in children [22], these shorter 6-mo pretomanid-containing regimens are not available for children <15 y of age. Despite this, expert opinion is that most children with RR/MDR-TB, especially those with non-severe disease, can achieve cure with shorter 6–9-mo, all-oral regimens [23, 24], but close monitoring of TB recurrence for at least 12 mo following treatment completion is essential.

## Treatment Regimens for RR/MDR-TB in Children

The 2022 WHO guidelines on treatment of RR/MDR-TB present two treatment approaches applicable to children aged <15 y: the standardised, 9–12-mo, all-oral regimen (with strict eligibility criteria), and individualized, all-oral regimens [18]. Both approaches avoid the use of toxic, painful injectable agents, which should not be used in children unless no other treatment options exist.

### Standardised, 9–12-mo RR/MDR-TB Regimen

This regimen consists of seven drugs with an intensive phase of 4–6 mo (duration dependent on smear-conversion by four months on treatment) of bedaquiline, moxifloxacin (or levofloxacin), clofazimine, ethambutol, ethionamide, high-dose isoniazid and pyrazinamide, and a continuation phase of 5–6 mo of clofazimine, moxifloxacin/levofloxacin, ethambutol and pyrazinamide. Two months of linezolid can be used as an alternative to 4–6 mo of ethionamide within this regimen [25]. The evidence upon which the recommendations for this regimen were based indicates that the composition and duration of the standardised regimen should not be modified beyond this [18].

Furthermore, treatment with this standardised regimen is restricted to RMR-TB and MDR-TB with no resistance to second-line drugs used in the regimen, particularly fluoroquinolones; access to rapid first- and second-line DST is essential. The regimen is not suitable for treatment of severe pulmonary or extrathoracic disease, or where both *inhA* and *katG* mutations confer resistance to isoniazid, or in people previously treated with second-line TB drugs for more than one month. These restrictions are especially important in countries like India where the proportion of patients with pre-XDR-TB is high [26]. Another disadvantage of this regimen for children is the inclusion of some potentially ineffective drugs that serve only to increase pill burden and carry additional risk for toxicity.

### Individualized RR/MDR-TB Regimens

**Fig. 1:**
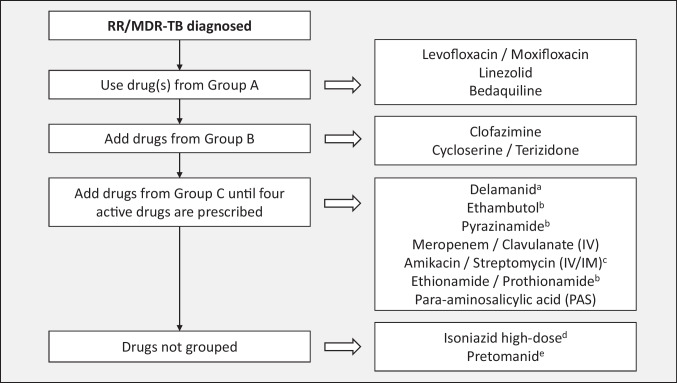
Building an individualized RR/MDR-TB regimen with 4-5 effective drugs, according to World Health Organization classification (Groups A-C) of second-line antituberculosis drugs (modified from previously published figure [4]). ^a^Delamanid is the preferred drug from group C, if a 4-5 effective drug regimen cannot be constructed from Group A and B drugs alone [11]. ^b^Resistance to ethambutol, pyrazinamide and ethionamide/prothionamide is common in many strains of RR/MDR-TB – susceptibility to these drugs must be confirmed to be considered effective. ^c^The aminoglycoside injectable agents should not be used in children unless no other drug options are available to construct an effective salvage regimen. ^d^High-dose isoniazid has shown efficacy in low-level isoniazid resistance (*inhA* mutation conferring resistance, in the absence of a *katG* mutation) [28]. However, cycloserine/terizidone may reduce isoniazid concentrations [14]. ^e^Pretomanid has not been studied in children <15 y of age – dose and safety in children is unknown. *RR/MDR-TB* Rifampicin-resistant/multidrug-resistant tuberculosis

This approach is preferable for treatment of RR/MDR-TB in children as the regimen can be tailored to include a minimum of four drugs considered to be effective for the individual child (Group A and B drugs are prioritized), and treatment duration can be shortened to 6–9 mo depending on disease severity, site of disease and extent of drug resistance [5, 11, 24, 27]. Four to five likely effective drugs, based on known DST results of the *M. tuberculosis* isolate from the child or source patient, as well as history of drugs used in prior failing regimens and therefore unlikely to be effective unless susceptibility is confirmed with recent DST results, are selected from the WHO drug groups to construct an initial regimen (Fig. 1) [4, 11, 14, 28]. As per WHO 2022 recommendations, amikacin should not be used for anyone under the age of 18 y, but may be considered in salvage regimens if no other options are available and susceptibility is confirmed [18]. Pediatric dosing recommendations are available for all antituberculosis drugs (Table 1), aside from pretomanid, which is not included in the WHO drug groups and is not yet recommended for children aged 0–14 y due to possible safety concerns [29]. Duration of bedaquiline and delamanid dosing is often restricted to six months; however, these drugs can be continued for longer with ongoing toxicity monitoring [27].

While individualized treatment is often preferred, even for children who are eligible for the standardised, 9–12-mo regimen [29], individualized regimens are specifically recommended in the following situations [4, 10]:

- MDR-TB plus resistance to any of the second-line drugs;
- Children who previously received second-line TB treatment for more than one month;
- All severe forms of extrathoracic TB, such as CNS TB, miliary TB and osteoarticular TB; treatment of CNS or miliary TB should include at least two effective drugs that penetrate the CSF well (Table 2);
- Children in whom the standardised 9–12-mo regimen is failing.

## Treatment Duration

**Fig. 2:**
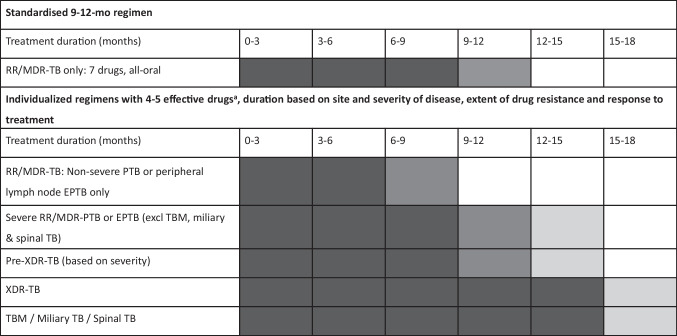
Suggested duration of treatment for RR/MDR-TB regimens in children. Black = definite treatment; dark grey = likely treatment; light grey = possible extension; white = no treatment. ^a^WHO group A and B drugs plus delamanid are prioritized [11]. *EPTB* Extrapulmonary tuberculosis, *Pre-XDR-TB* Pre-extensively drug-resistant tuberculosis, *PTB* Pulmonary tuberculosis, *RR/MDR-TB* Rifampicin-resistant/multidrug-resistant tuberculosis, *TBM* Tuberculosis meningitis, *XDR-TB* Extensively drug-resistant tuberculosis

Treatment duration with individualized RR/MDR-TB regimens in children is usually based on site and severity of disease and extent of *M. tuberculosis* drug resistance (Fig. 2). Results from the SHINE trial [15] showed that children with non-severe drug-susceptible TB disease can be successfully treated with a shorter (4-mo) treatment duration. Therefore, children with non-severe RR/MDR-TB disease are also likely to be successfully treated with shorter (6–9-mo) regimens containing at least 4 effective drugs; those with more severe or extensively resistant TB disease are likely to require treatment for 9–12 mo or longer (15–18 mo for miliary/CNS/spinal RR-TB) (Fig. 2). Clinicians may also extend treatment in individuals with slow or inadequate clinical, radiological and/or microbiological treatment response or immunological compromise.

There is no specified intensive or continuation phase for pediatric RR/MDR-TB regimens and most drugs should be continued throughout treatment, if possible, unless limited by toxicity or intolerance. Bedaquiline and delamanid are usually given for six months only but may be extended if other effective drug options within the regimen are limited [27]. Linezolid is usually only given to children for two months; however, some children may benefit from linezolid for the full duration of treatment if tolerated.

## RR/MDR-TB and HIV

In children with RR/MDR-TB and living with HIV, early initiation of antiretroviral therapy is indicated, except in CNS RR/MDR-TB where antiretroviral therapy initiation should be delayed for at least one month. In general, drug-drug interactions are fewer than with drug-susceptible TB treatment, as the rifamycins are excluded from RR/MDR-TB regimens. Of note, bedaquiline concentration is reduced by 50 percent if co-administered with efavirenz [30], therefore antiretroviral therapy regimens should be amended accordingly.

## Treatment Adherence and Drug Adverse Effects

In addition to inappropriate treatment regimens, factors affecting adherence to treatment may be associated with unsuccessful RR/MDR-TB treatment outcomes in children [31]. Adherence interventions should target caregivers and healthcare workers who are responsible for administration of medication to children [32, 33]. Both the caregiver(s) and the child, as appropriate, should be counselled properly at every follow-up visit about RR/MDR-TB, preferred treatment options, treatment duration and possible adverse effects [34]. Caregivers and healthcare workers/treatment supporters require education, training and ongoing support to correctly identify the different TB medications and how to prepare and administer specific doses for an individual child. Dosages may require adjustment for weight gain during treatment. Persons dispensing the TB drugs to the caregivers for administration to the child at home are responsible for checking and ensuring that the correct formulations at the correct doses are provided, especially if there is more than one formulation for a specific drug. Preparation and administration of RR/MDR-TB medications can be complicated, particularly for very young children, and caregivers may require ongoing support and reaffirmation throughout treatment.

Some children are hospitalized for clinical reasons during TB treatment, while some are admitted due to the absence of a reliable caregiver [35]. Preferably, clinically stable children should be treated out of hospital, provided a reliable person (caregiver, healthcare worker or treatment supporter) is available and willing to supervise treatment administration. Directly observed therapy (DOT) is widely advocated, but alternative methods such as video observed treatment (VOT) or digital adherence technologies (DAT) can also facilitate treatment monitoring [34].

RR/MDR-TB often occurs in financially challenged families, and may affect more than one family member, which could lead to further financial strain and food insecurity. Therefore, patients and families may need nutritional and/or financial support to successfully complete their treatment.

Children must be followed up at least monthly to assess clinical progress and monitor for potential adverse effects of second-line antituberculosis drugs (Table 2); some adverse effects are severe and may require drug changes or dose reduction (within the therapeutic dose range), while other less severe adverse effects may lead to non-adherence if not acknowledged and effectively addressed. Ototoxicity associated with injectable aminoglycosides is common, irreversible, and interferes with a child’s optimal development, and these agents should be avoided unless absolutely necessary. Some adverse effects, such as peripheral neuropathy, arthralgia, and changes in vision, are challenging to identify and assess in young children; regular and thorough clinical history and examination is the key to identifying problems early. Discontinuation of a drug may be necessary in some situations – the severity of disease, extent of drug resistance and clinical response to treatment up to the time of the adverse effect should be considered when evaluating treatment options, i.e. continuation of the regimen without the offending agent, simple substitution of the drug with another effective agent (avoid if the regimen is failing), or change in the entire regimen (in the case of a failing regimen).

## Treatment Outcomes

Most children who are diagnosed with either presumed or confirmed RR/MDR-TB, and started on appropriate treatment regimens, generally have good outcomes. Several retrospective and prospective studies among children have shown high rates (approximately 80–90%) of treatment success (cure and treatment completion) [36–38]. However, outcomes among children with undiagnosed and untreated TB remain dire [39]. In a modelling study, Dodd et al. estimated that, with universal household screening of children in contact with RR/MDR-TB adults in 2019, 227,000 children (95% uncertainty interval [UI]: 205,000–252,000) younger than 15 y globally would have been screened, and 2,350 (95% UI 1,940–2,790) tuberculosis deaths averted. If all the child household contacts of infectious RR/MDR-TB patients received TB preventive therapy (TPT) with levofloxacin, 5,620 incident tuberculosis cases (95% UI 4,540–6,890) and an additional 1,240 deaths (95% UI 970–1,540) would have been prevented [40].

## Way Forward

Prevention of RR/MDR-TB in children should be a public health priority, through pro-active contact tracing of children exposed to infectious RR/MDR-TB patients, clinical assessment to exclude TB disease and prompt initiation of TPT. Several randomised controlled studies are ongoing to find effective single-drug TPT options for RR/MDR-TB. Current recommendations are to use levofloxacin with or without an additional drug to which the source patient’s *M. tuberculosis* strain is susceptible, for six months’ duration [11].

The novel agents, bedaquiline and delamanid, are widely available for all ages and weight groups. The availability of these effective drugs eliminates the need for injectable agents in RR/MDR-TB regimens. Child-friendly formulations have been developed for both of these drugs and should be used whenever possible; however, the adult formulations of both bedaquiline and delamanid when crushed and dispersed in water have shown bioequivalence to tablets swallowed whole in adults [41, 42]. Several RR/MDR-TB treatment-shortening trials have shown efficacy of 6-mo regimens in adults; following this, studies of 6-mo, all-oral RR/MDR-TB regimens for children are being planned. However, despite these considerable therapeutic advances, systematic screening of children exposed to infectious RR/MDR-TB patients, appropriate preventive management and early detection of TB disease remain the biggest challenges for pediatric RR/MDR-TB control in high TB burden countries worldwide.
